# Correction: Mid-infrared spectroscopy and microscopy of subcellular structures in eukaryotic cells with atomic force microscopy – infrared spectroscopy

**DOI:** 10.1039/c9ra90066g

**Published:** 2019-09-25

**Authors:** Luca Quaroni, Katarzyna Pogoda, Joanna Wiltowska-Zuber, Wojciech M. Kwiatek

**Affiliations:** Department of Experimental Physics of Complex Systems, Institute of Nuclear Physics, Polish Academy of Sciences PL-31342 Kraków Poland luca.quaroni@uj.edu.pl; Department of Physical Chemistry and Electrochemistry, Faculty of Chemistry, Jagiellonian University PL-30387 Kraków Poland

## Abstract

Correction for ‘Mid-infrared spectroscopy and microscopy of subcellular structures in eukaryotic cells with atomic force microscopy – infrared spectroscopy’ by Luca Quaroni *et al.*, *RSC Adv.*, 2018, **8**, 2786–2794.

In the article the assignment of two IR absorption bands, at 3010 and at 3070 cm^−1^ has been confused by us in the text, resulting in two incorrect statements. The misstatement does not change any of the conclusions of the work, and can be corrected by restating the following sentences.

The following sentence (p. 2790, column 1, line 10):

A weak but sharp band can be seen at 3010 cm^−1^, corresponding to the stretching mode of C–H bonds on unsaturated C=C bonds in acyl chains.

must be corrected to:

A weak but sharp band can be seen at 3010 cm^−1^, corresponding to the stretching mode of C–H_3_ bonds on choline headgroups.

In addition, the following sentence (p. 2792, column 1, line 25):

Observation of a sharp band at 3010 cm^−1^ indicates that at least part of the acyl chains have unsaturated C=C bonds.

must be changed to:

Observation of a band at 3070 cm^−1^, corresponding to the stretching mode of C–H bonds on unsaturated C=C bonds in acyl chains, indicates that at least part of the acyl chains have unsaturated C=C bonds.

The Royal Society of Chemistry apologises for these errors and any consequent inconvenience to authors and readers.

## Supplementary Material

